# Intraoperative localization of the marginal mandibular nerve: a landmark study

**DOI:** 10.1186/s13104-015-1322-6

**Published:** 2015-08-27

**Authors:** Khalid AL-Qahtani, Alex Mlynarek, Jon Adamis, Jeffery Harris, Hadi Seikaly, Tahera Islam

**Affiliations:** Division of Otolaryngology-Head and Neck Surgery, University of Alberta, Edmonton, Canada; Department of Otolaryngology-Head and Neck Surgery, College of Medicine, King Saud University, Riyadh, Kingdom of Saudi Arabia; College of Medicine and Research Center, King Saud University, Riyadh, Saudi Arabia; King Abdul Aziz University Hospital, King Saud University, PO Box no-245, Riyadh, 11411 Kingdom of Saudi Arabia

**Keywords:** Marginal mandibular nerve (MMN), Angle of the mandible, Inferior border of the mandible, Subplatysmal flap, Neck dissection, Oral cancer

## Abstract

**Background:**

Identification and preservation of the marginal mandibular nerve (MMN) remains an important step in otolaryngology procedures. Current publications place the MMN at least 1 cm below the mandible. This study will evaluate the accuracy of the method of determining the surgical location of this branch of the facial nerve in vivo.

**Methods:**

MMN were examined in 52 consecutive otolaryngology patients. Using a validated landmarking scheme, distances were measured from the inferior edge of the mandible and the lowest point of the nerve. A comparison of 33 nerves pairs was undertaken. Effect of patient’s age was analysed.

**Results:**

Eighty five nerves were landmarked. The mean position of the nerve was 0.2–3.4 mm higher than the margin of the mandible. There were no significant difference in position with respect to age and left versus right comparisons.

**Conclusion:**

The marginal mandibular nerve (MMN) is significantly higher than previously published. The location of the nerve on the right does not correlate with the left. Location of the nerve does not correlate with patient’s age.

## Background

The facial or VII cranial nerve is a mixed nerve composed of both motor and sensory branches and is responsible for the motor innervations of facial expression muscles, lachrymal secretion and partial control of the gustatory sensation [1]. The facial nerve enters the posteromedial surface of the parotid gland, crosses superficial to the external carotid artery and the retromandibular vein, divides into a number of branches that emerge separately from the gland and passes to supply the muscles of facial expression. The temporal, zygomatic, buccal, marginal mandibular, and cervical are the five major branches of the facial nerve, with the marginal mandibular branch being at particular risk during surgical procedures in the submandibular region [2–6] and rhytidectomy/liposuction [7].

Identification and preservation of the marginal mandibular nerve remains a critical step in otolaryngologic, cosmetic and oncologic head and neck surgery. Iatrogenic injury to the marginal mandibular branch (MMB) during surgery of the neck often results in disorders of facial expression and has been an important reason of medicolegal actions [8]. Injury to the marginal mandibular nerve is present as an adverse outcome in many surgical procedures. The resultant cosmetic deficit, manifesting lower lip asymmetry and imbalance, is readily noticeable especially during opening of the mouth [9].

The marginal mandibular branch supplies the muscles of facial expression that pull and close the angle of the mouth and pulls the lower lip downward (depressor labii inferioris and depressor anguli oris). The nerve passes along the inferior border of the mandible, often looping down into the neck, deep to the platysma and depressor anguli oris [10]. Touré et al. [11] reports a case where the marginal mandibular branch was situated 17.5 mm from the inferior border of the mandible. Due to its location, this branch can occasionally be damaged during cervical surgeries, parotidectomies, open reductions of mandibular angle fractures, rhytidoplasties and other surgeries confined to the submandibular region [12]. Also this higher incidence of injury to the marginal mandibular branch may be related to the proportionately greater number of operations in the region of the mandible, but must also be related to the lack of an accurate description of the course of this nerve in the anatomical textbooks [13, 14]. This is why the knowledge of the course and anatomic relations of the marginal mandibular nerve in the upper neck is important in avoiding injury [15]. Unrecognized or inadvertent injury results in a significant cosmetic deformity (flattening and inversion of the ipsilateral lip) that is very difficult to correct [8].

Distortion of the position of the facial nerve due to parotid tumors will also affect the position and anatomy of the marginal mandibular branch. Perhaps one of the most challenging situations is associated with a displaced facial nerve in large or vascular tumors. It is important for the surgeon to maintain a hemostatic field to identify the nerve accurately. The marginal mandibular branch is used frequently as a guide to the main trunk of the facial nerve, but clear guidelines for locating the marginal mandibular branch are often lacking [8].

Although the intraparotid anatomy of the facial nerve has been very well documented, the surgical approaches to the peripheral, extraparotid branches of the facial nerve have not been described as accurately. The direction followed by the facial nerve branches beyond their emergence from the ventral, cephalic and caudal borders of the parotid gland up to the facial muscles has been studied by several anatomists, but so far, no consistent description of them has been given [16].

At the University of Alberta, over 350 procedures performed annually that requires the identification of the marginal mandibular nerve. The positions of the marginal mandibular nerve were frequently observed to be higher than the published literature. So our hypothesis was the marginal mandibular nerve is located significantly higher than previously published. The objective of this study is to assess the position of the marginal mandibular nerve in relation to the inferior border of the mandible.

## Methods

**Fig. 1:**
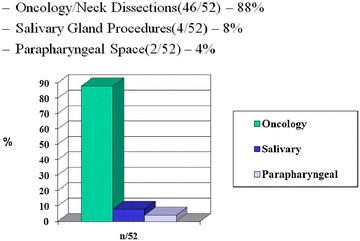
Types of surgery

The study was approved by the human research ethics board at the University of Alberta, and informed consent was obtained from each patient for use of their data within the prospective in vivo study. Fifty two consecutive patients were enrolled between September 2005–March 2006. Our exclusion criteria were 1-tumor involvement of the submandibular region and/or 2-previous irradiation. We identified 85 nerves. The types of surgeries performed were: oncology/neck dissections (46/52)—88 %, salivary gland procedures (4/52)—8 %, parapharyngeal space (2/52)—4 % (Fig. 1).

All patients were positioned with a roll under the shoulders to maintain extension of the neck. Skin incisions in the upper neck were placed in skin creases 2–4 cm below the lower border of the mandible. In general, skin flaps were elevated with sharp dissection in a sub-platysmal plane. When level I was dissected an attempt was made to identify the marginal mandibular nerve using standard landmarks [5, 8, 17]. This generally involved observation of the nerve coursing superficial to the anterior facial vein.

**Fig. 2:**
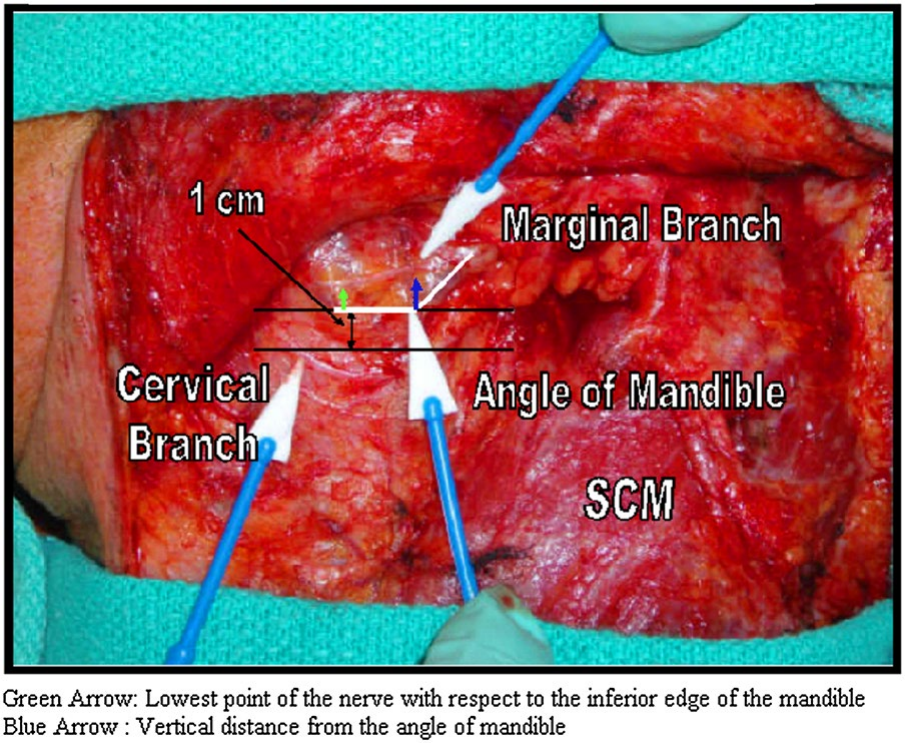
Reference lines and measurements

The MM and cervical nerves were carefully dissected in a sub-platysmal plane. The posterior limit was insertion into the tail of parotid and anterior limit superior course above mandible. Two constant reference lines were created Inferior border of the mandible and 10 mm (1 cm) below the mandible (Fig. 2).

The measurements of 1-vertical distance from the angle of the mandible (blue arrow) and 2-lowest point of the nerve with respect to the inferior edge of the mandible (green arrow) were obtained (Fig. 2). Variable intensity stimulator was used to stimulate both the marginal and cervical branches. MMN was identified by observing the mentalis muscle contraction, twitching of lower lip. The cervical branches were then cut. Evaluation of the marginal nerve was then conducted in the ward postoperatively.

### Statistical analysis

Results were tabulated and analyzed statistically by using:

1. One-sample t test.
2. Paired samples t test.
3. Tests of agreement and association.
  Kappa and lambda statistics.
4. Nerve position association with age.
  One-way ANOVA, Pearson correlation and linear regression.

## Results

Fifty two patients were enrolled in the study, 36 males and 16 females. 85 Nerves were tested, 47 right and 38 left. Mean age was 57.6 years (range 24–87 years). 33 Patients with bilateral measurements.

Marginal mandibular nerve location relative to the inferior border of mandible was measured (Table 1). We were comparing the locations of the nerve (the mean nerve position, the lowest, and the highest position) to the angle and the lowest border of the mandible in the right and left side (Table 1). The main nerve position in the right side was 2.7 mm above the right angle and 0.2 mm above the right lowest point, while in the left side the mean nerve position was 3.4 mm above the angle and 1.3 mm above the lower point. On the other hand the lowest nerve position in the right hand side was 10 mm below the angle of the mandible and to the lowest point of the mandible as well, while in the left side, the lowest point was 6 mm below the angle of the mandible and 10 mm below the lower point. Also, the location of the highest point of the nerve compared to the angle and the lowest point of the mandible in both sides, which showed that in the right side the highest point is 20 mm above the angle of the mandible and 13 mm above the lowest point of the mandible. In the left side, the highest point was 13 mm above the left angle and 10 mm above the lowest point of the mandible.

**Table 1:** Marginal mandibular nerve location relative to the inferior border of mandible

	Mean nerve position^a^	Lowest	Highest
Right angle	2.7 Above	10 Below	20 Above
Right lowest	0.2 Above	10 Below	10 Above
Left angle	3.4 Above	6 Below	13 Above
Left lowest	1.3 Above	10 Below	10 Above

^a^All units are in millimeters (mm)

Also, MM nerve position relative to the test value of 10 mm below the mandible was measured (Table 2), and we measured the mean difference, P value and 95 % CI in relation to the angle on the mandible and lowest point in right and left side. The mean difference of the position of the MMN and the angle of the mandible and the lowest point of the mandible were measured, and showed that the mean difference to the right angle 12.723 mm, to the right lowest point is 10.149, to the left angle is 13.395 mm, and to the left lowest point is 11.342 mm. The p value was measured and it was <0.001 (statistically significant). Also, the 95 % confidence interval (CI) in relation to the four points were measured and showed 11.19–14.25 to the right angle, 9.09–11.21 to the right lowest point, 11.87–14.92 to the left angle, and 9.92–12.76 to the left lowest point.

**Table 2:** MM nerve position relative to the test value of 10 mm below the mandible

	Mean difference^a^	p value	95 % CI
Right angle	12.723	<0.001	11.19–14.25
Right lowest	10.149	<0.001	9.09–11.21
Left angle	13.395	<0.001	11.87–14.92
Left lowest	11.342	<0.001	9.92–12.76

^a^All units are in millimeters (mm)

Right versus left comparison relative to inferior border of mandible was done (Table 3). Thirty three of 52 patients (64 %) with bilateral MM measurement. We measured the mean Rt and Lt MMN relative to the angle and lowest point. Also, the difference and the p value were measured. The kappa was 0.106 and lambda was 0. The comparison for the angle was 2.0 mm above the mean Rt MMN, 3.3 mm above the mean Lt MMN, the difference was 1.3 mm, and the p value was 0.12 (not statistically significant). While the comparison for the lowest point was 0.8 mm below the mean Rt MMN, 1.2 mm above the mean Lt MMN, the difference was 2.0 mm, and the p value was 0.04 (statistically significant).

**Table 3:** Right versus left comparison relative to inferior border of mandible 33 of 52 patients (64 %) with bilateral MM nerve measurements

	Angle	Lowest
Mean Rt MMN	2.0 Above	0.8 Below
Mean Lt MMN	3.3 Above	1.2 Above
Difference	1.3	2.0
p value	0.12 (NS)	0.04

^a^All units are in millimeters (mm), κ = 0.106, λ = 0

**Fig. 3:**
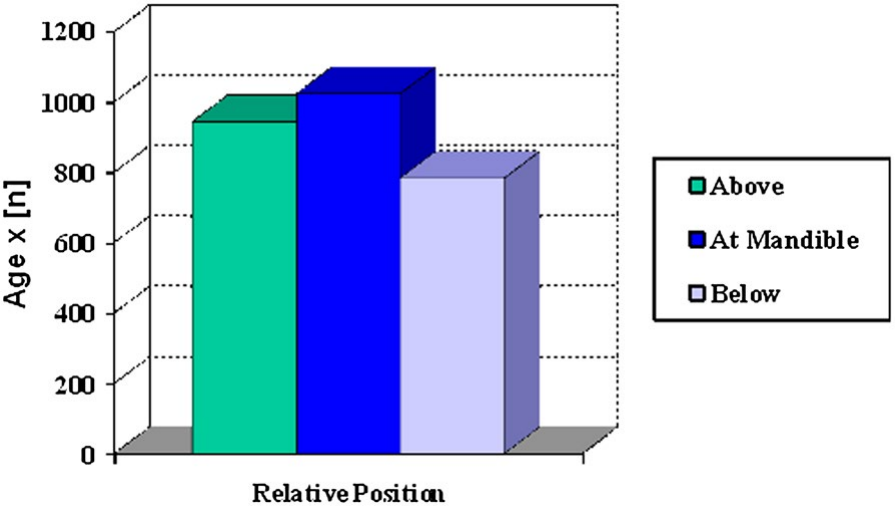
Effect of age

Correlation and regression analysis of age to lowest position of MM nerve was done (Fig. 3). The ANOVA mean comparisons was NS. The correlation and regression analysis of age to lowest position of MM nerve as follows:

- Right MMN: Pearson correlation coefficient: 0.11. Linear regression of age: p = 0.46 (NS).
- Left MMN: Pearson correlation coefficient: 0.24. Linear regression of age: p = 0.15 (NS).

## Discussion

The marginal mandibular branch of the facial nerve can be difficult to locate in the process of performing a neck dissection, or for any procedure in the upper neck. Careful attention to the anatomic relationships is needed to allow safe identification and preservation of this important structure. During a neck dissection, complete clearance of nodal tissue is often required that requires a sound anatomical knowledge of the nerve.

Current publications describe the position of this facial nerve branch as “about 1 cm in front of and below the angle of the mandible” [18], then “the nerve curves downwards below and in front of the mandible across the facial vessels about 1 finger’s breadth below the mandible” [19]. However, at the University of Alberta, over 350 procedures are performed annually that necessitate the identification of the marginal mandibular nerve. The nerve was frequently discovered to be passing in higher than published position of the marginal mandibular nerve. The anatomy of the marginal mandibular nerve in its vulnerable position in the upper neck is variable between patients. The course and position of the nerve also varies with the position of the head and downward traction on the investing layer of cervical fascia. The dynamic nature of the nerve was evident in this study. This explains, in part, the discrepancies between cadaver studies and clinical observations. Cadaver tissue is contracted and relatively immobile. Of interest, when fresh as opposed to embalmed cadaver material is studied, the nerve is consistently reported below the lower border of the mandible. The comparison between the result of different previous studies is summarized in Table 4. In a series of 22 fresh cadavers Savary et al. [20] observed some branches as low as 3–4 cm below the lower border of the mandible.

**Table 4:** Relation of MMN with inferior border of mandible and facial artery, comparison of previous publications

References	Subject	Reference to facial artery	Reference to lower border mandible	
			Above	Below
Dingman and Grabb [5]	Cadaver	Ant to	100 %	0
		Post to	81 %	19 %
Wang et al. [12]	Cadaver	Ant to	90 %	10 %
		Post to	67 %	33 %
Savary et al. [20]	Cadaver	Ant to	^a^	27 %
		Post to	^a^	63 %
Nason et al. [15]	Patient		^a^	64 %
Nelson and Gingrass [17]	Cadaver			100 %
Baker and Conley [21]	Patient			Almost 100 %
Woltmann et al. [29]	Cadaver		57.70 %	43.30 %
Ziarah and Atkinson [25]	Cadaver		47 %	53 %

*ant* anterior,* post* posterior

^a^Not reported

Nelson and Gingrass [17] noted that the marginal mandibular nerve was well below the inferior border of the mandible in almost every instance in fresh cadaver specimens and clinical dissections. Both these reports recommended cervical incisions several centimeters below the inferior border of the mandible. Nason et al. [15] confirms the consistent course of the marginal nerve below the inferior border of the mandible.

The observation by Baker and Conley [21] that the nerve is drawn downward with the extension of neck is confirmed. A cervical incisions 2 cm below the lower border of mandible will place the nerve at risk in a significant number of patients. Detailed anatomic dissections [5, 8, 17] show that there are often multiple contributions to the marginal mandibular nerve, and that it can be difficult to discern between mandibular and platysmal branches. Dingman and Grabb [5] noted that in many specimens fine branches could be seen running along the lower border of the mandible, some up to 2 cm below it. They stated that these branches terminated in and innervated the platysma in all specimens. Nelson and Gingrass [17] argued that these same branches, when followed anteriorly in fresh cadaver specimens and in clinical practice, ascended over the lower border of the mandible to innervate specific lip depressors. They contend that these branches should be identified as mandibular and not cervical branches. Skandalakis et al. [22] describes an anterior ramus of the cervical division of the facial nerve that joins the mandibular ramus to contribute to the innervations of the lower lip. There are also observations that the platysma, innervated by the cervical division of the facial nerve, contributes to depression of the lower lip [6]. Division of the cervical branch of the facial nerve or the platysma can result in a pseudo-paralysis of the marginal mandibular nerve that usually recovers spontaneously. From a practical point of view there is usually a single identifiable nerve ramus coursing superficial to the facial veins that results in asymmetry of the lower lip if divided. Stern [8] indicates that if the nerve does not course above the inferior border of the mandible within 2 cm of the facial vessels it is not the marginal mandibular nerve. A low cervical incision with a ‘nonvisualization’ approach to the marginal mandibular nerve is supported [23, 24].

Ziarah and Atkinson [25] dissected 76 human facial halves and found marginal mandibular nerve distances to the inferior margin of the mandible no greater than 1.2 cm. On the other hand, Wang et al. [12] found values varying from 0 to 3 cm, and in 95.64 % the nerve passed 0–2 cm away below the inferior margin of the mandible.

In a study by Dingman and Grabb [5], 100 facial halves were dissected with specific attention to the course of the marginal mandibular branch in relationship to the lower edge of the mandible. Posterior to the facial artery, the mandibular branch mostly remained superior to the inferior border of the mandible. However in 19 % one or more rami of the marginal mandibular branch formed a downward arc, whose lowest point extended 1.0 cm below the inferior border of the mandible.

Potgieter et al. [26] indicates a range between −6.2 and 5.1 mm (negative value ¼ inferior to landmark; positive value ¼ superior to landmark) of the MMB to the angle of the mandible and a range between −6.9 and 7.4 mm of the nerve to a point just anterior to the facial artery on the inferior border of the mandible. This suggests a slightly lower than the reported position of the MMB posterior to the facial artery when compared to the work by Dingman and Grabb [5], and almost as low as suggested by Baker and Conley [21].

Peterson and Johnston studied 100 patients undergoing parotidectomy and found that the marginal mandibular branch can be located near the angle of the mandible at a point 4.0–4.5 cm from the attachment of the ear lobe [27]. Baker and Conley [21] reported that in their experience with parotidectomies, the marginal mandibular branch was located 1–2 cm below the inferior border of the mandible in almost every instance. In this study, the difference between their report and that of Dingman and Grabb was explained by the fact that their patients were surgical cases with head rotated and extended, whereas Dingman and Grabb had studied preserved cadavers [5].

In the present study, we describe the accurate course and localization of the marginal mandibular branch by using the ramus of the mandible and facial artery as certain anatomical landmarks. Both of these landmarks can be felt easily by palpation in humans and therefore, they have a practical importance for the surgeon. Marginal mandibular nerve location relative to the inferior border of mandible was measured (Table 1).

In our study, the main nerve position in the right side was 2.7 mm above the right angle and 0.2 mm above the right lowest point, while in the left side the mean nerve position was 3.4 mm above the angle and 1.3 mm above the lower point. On the other hand the lowest nerve position in the right hand side was 10 mm below the angle of the mandible and to the lowest point of the mandible as well, while in the left side, the lowest point was 6 mm below the angle of the mandible and 10 mm below the lower point. Also, the location of the highest point of the nerve compared to the angle and the lowest point of the mandible in both sides, which showed that in the right side the highest point is 20 mm above the angle of the mandible and 13 mm above the lowest point of the mandible. In the left side, the highest point was 13 mm above the left angle and 10 mm above the lowest point of the mandible. The mean difference of the position of the MMN and the angle of the mandible and the lowest point of the mandible to the right angle 12.723 mm, to the right lowest point is 10.149, to the left angle is 13.395 mm, and to the left lowest point is 11.342 mm. The p value was <0.001 (statistically significant). Also, the 95 % confidence interval (CI) in relation to the four points showed 11.19–14.25 to the right angle, 9.09–11.21 to the right lowest point, 11.87–14.92 to the left angle, and 9.92–12.76 to the left lowest point.

The anatomic changes that occur in the aging face are delineated. With an adequate understanding of the anatomic changes that occur with aging, rhytidectomy can be approached as a reconstructive procedure, restoring facial soft tissue to its original state and location [28]. In clinical experiences with parotidectomies and radical neck dissections, the mandibular branch of the facial nerve has been 1–2 cm below the lower border of the mandible in almost every instance. In some individuals with lax and atrophic tissues, the branches were even 3–4 cm below [21].

However in the present study, right versus left comparison in relation to inferior border of mandible was done (Table 3). 33 Of 52 patients (64 %) with bilateral MM measurement. We measured the mean Rt and Lt MMN relative to the angle and lowest point. Also, the difference and the p value were measured. The kappa was 0.106 and lambda was 0. The comparison for the angle was 2.0 mm above the mean Rt MMN, 3.3 mm above the mean Lt MMN, the difference was 1.3 mm, and the p value was 0.12 (not statistically significant). While the comparison for the lowest point was 0.8 mm below the mean Rt MMN, 1.2 mm above the mean Lt MMN, the difference was 2.0 mm, and the p value was 0.04 (statistically significant).

Correlation and regression analysis of age to lowest position of MM nerve was done (Fig. 3). The ANOVA mean comparisons was NS. The correlation and regression analysis of age to lowest position of MM nerve as follows:

- Right MMN: Pearson correlation coefficient: 0.11. Linear regression of age: p = 0.46 (NS).
- Left MMN: Pearson correlation coefficient: 0.24. Linear regression of age: p = 0.15 (NS).

Significant difference in position with respect to age and left versus right comparisons was not found.

Irrespective of the site of the skin incision, skin flaps should be carefully elevated in a plane immediately deep to the platysma and superficial to the investing layer of deep cervical fascia and marginal mandibular nerve. It is not the level of the skin incision that is important but the level of transection of the investing layer of cervical fascia. Knowledge of the dynamic and variable location of the marginal mandibular nerve relative to the inferior border of the mandible is useful in this regard. The decision to visualize the nerve needs to be individualized. If it is deemed necessary to divide the cervical fascia within 3–4 cm of the inferior border of the mandible it is the authors’ opinion that an attempt should be made to visualize the course of the nerve. The nerve is then preserved or sacrificed based on the oncologic objectives of the procedure. Electrical cautery should be used sparingly, and attention should be directed to avoiding traction or pressure injury from retractors [15].

However, we recommend a novel surgical approach to the safe identification and preservation of the marginal mandibular nerve:

Incision has to be placed 4 cm below inferior border of mandible. Elevation of sub-platysmal plane has to be performed till above the mandible, leaving behind the superficial fascia along with the facial lymphnodes. Meticulous dissection of superficial fascia has to be performed at mandible parallel to the course of the nerve to locate the MMN. Following the identification of MMN the facial lymph nodes (nodes of star) has to be dissected, sparing the MMN in case of malignancy, specially oral cavity cancer. This technique creates the field to dissect the nodes more aggressively simultaneously ensuring preservation of MMN, thus should be the preferable approach for oncological cases. Most of the patients in this study suffered from head and neck malignancy and none of them had any metastatic recurrence in the neck nodes. We advocate this technique for both malignant and non-malignant situations.

There were some limitations:

Quantitatively landmarking of the cervical branch. Also, the analysis of extent and etiology of disease, physical characteristics (neck circumference), and intra-operative positioning of patients.

## Conclusions

1. The in vivo course of the marginal mandibular nerve is significantly higher than previously published.
2. The location of the nerve on the right does not correlate with the left.
3. Location of the nerve does not correlate with patient’s age.

## Abbreviations

MMN: Marginal mandibular nerve
MMB: Marginal mandibular branch
CI: Confidence interval
